# Lymphodepletion chemotherapy revitalizes chimeric antigen receptor T cells contributing to regression of relapsed B-cell lymphoma: A case report

**DOI:** 10.1097/MD.0000000000022510

**Published:** 2020-10-23

**Authors:** Zuyu Liang, Hao Zhang, Mi Shao, Qu Cui, Zhao Wu, Lei Xiao, He Huang, Yongxian Hu

**Affiliations:** a Bone Marrow Transplantation Center, The First Affiliated Hospital, School of Medicine; b Hematology Institution, Zhejiang University, Hangzhou; c Department of Hematology, Beijing Tiantan Hospital, Capital Medical University, Beijing; d Innovative Cellular Therapeutics Co., Ltd., Shanghai, China.

**Keywords:** chemotherapy, chimeric antigen receptor T cells, cytokine release syndrome

## Abstract

**Introduction:**

Chimeric antigen receptor T cells (CAR-T) targeting CD19 have shown great potential for treatment of B-cell malignancies. For those patients who can not achieve complete remission (CR) or suffer from relapse after CAR-T therapy, further therapeutic strategies still remain elusive. Whether existing CAR-T cells can revitalize in vivo and eradicate tumor cells is still unknown.

**Patient concerns:**

We report a case of diffused large B-cell lymphoma patient who had achieved CR after CD19 targeted CAR-T therapy but relapsed after 5 months.

**Diagnosis:**

Five months after CAR-T cell infusion, the patient was confirmed a relapse by follow-up PET/CT scan and a mass biopsy. Flow cytometry showed a dramatically decreased percentage of CAR-T cells in peripheral blood (PB).

**Interventions:**

A second anti-CD19 CAR-T therapy was planned with deliberation. Firstly, the patient received lymphodepletion chemotherapy with fludarabine (25 mg/m^2^, d1–d3) and cyclophosphamide (500 mg/m^2^ d2–d3).

**Outcomes:**

After fludarabine and cyclophosphamide (FC) lymphodepletion chemotherapy, pre-existing CAR-T cells were revitalized and the patient developed grade 2 cytokine release syndrome (CRS) contributing to the regression of relapsed B-cell lymphoma.

**Conclusions:**

This case suggested that FC chemotherapy could revitalize CAR-T cells contributing to the regression of relapsed B-cell lymphoma. Nevertheless, further researches are required in the future as this report described only a single case.

## 1 Introduction

Adoptive T-cell therapy with genetically modified chimeric antigen receptor (CAR) targeting CD19 (CART19) has shown great potential for treatment of B-cell malignancies.^[1–3]^ For patients with relapsed/refractory B-cell NHL, an overall response rate is about 70% to 80% and the complete remission (CR) rate is about 40% to 50% after CART19 treatment.^[4]^ Thus, the efficacy of chimeric antigen receptor T cell (CAR-T) strategy for B-cell NHL still needs substantial improvement.

Before CAR-T cell infusion, patients always receive lymphodepletion chemotherapy, usually consisting of cyclophosphamide (Cy) alone or Cy combined with fludarabine (FC). Lymphodepletion chemotherapy could reduce endogenous lymphocyte numbers, thereby increase availability of homeostatic cytokines that promote survival of transferred T cells.^[5]^ CAR-T cells exert cytotoxicity against tumor cells. Simultaneously, cytokine release syndrome (CRS) may occur, due to effects induced by binding of CAR-T cell receptor to matched antigens and subsequent activation of bystander immune cells, as well as non-immune cells. The risk of CRS is supposed to be influenced by factors including tumor burden, dosing and type of infused CAR-T cells, and lymphodepletion regimen, etc.^[6]^ A higher incidence of CRS was observed after lymphodepletion with Cy or fludarabine,^[7]^ while several studies demonstrated a direct association between the severity of CRS and clinical responses.^[8,9]^ Based on these findings, CRS is accordingly considered the most important biomarker of CAR-T cell activation and was positively correlated with therapeutic efficacy and prognosis.

In recent studies, the complete remission (CR) rate of CAR-T therapy for diffused large B-cell lymphoma (DLBCL) was about 50% and a relapse rate about 10% was observed. For those who failed to achieve CR or suffer from relapse after CAR-T therapy, further therapeutic strategies still remain elusive. Available modalities may include salvage chemotherapy or a second CAR-T infusion, unfortunately, the prognosis of neither treatment was poor. Re-boosting previously-infused CAR-T would be somehow an option, nevertheless, whether those CAR-T cells could be revitalized in vivo to eradicate tumor cells is unknown. Herein, for the first time we report a case of diffused large B-cell lymphoma patient who achieved CR by CART19 treatment but relapsed at 5 months. In the following treatment, pre-existing CAR-T cells were revitalized with a grade 2 CRS after FC lymphodepletion chemotherapy, and finally induced tumor regression for a second time.

## 2 Case presentation

**Figure 1:**
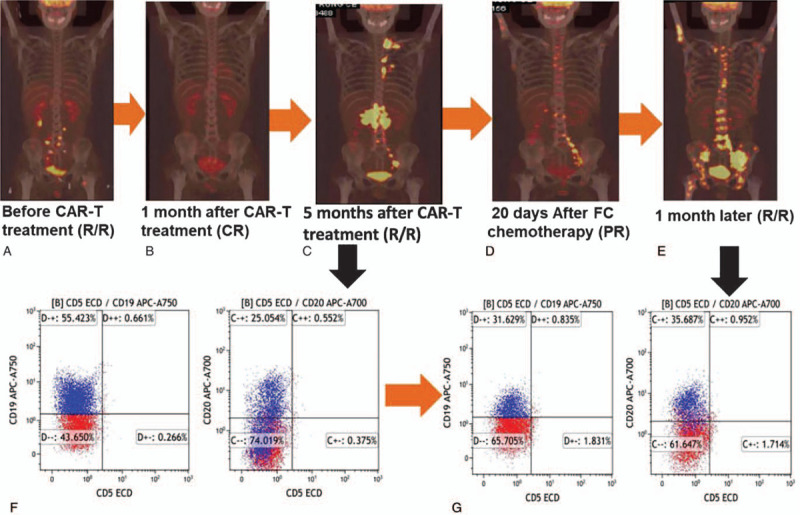
The patient prognosis after CAR-T treatment. The patient with refractory/relapsed lymphoma (A) achieved complete remission 1 month after CAR-T treatment (B) but relapsed 5 months later (C) with CD19 high expression (F). After FC lymphodepletion chemotherapy, CAR-T cells were revitalized and the patient developed grade 2 cytokine release syndrome (CRS) contributing to partial remission 20 days later (D) but relapsed again (E) with CD19 dim expression (G). CR = complete remission; PR = partial remission; R/R = refractory/relapsed.

The patient had been described previously due to delayed terminal ileal perforation following CAR-T cell therapy.^[10]^ Briefly, a 36-year-old man, diagnosed as DLBCL in 2003, initiated a 6-cycle therapy of EPOCH and achieved CR. The disease relapsed in June 2014. Then after a 4-cycle therapy of R-CHOP and a 2-cycle R-IVAC the patient achieved CR2. In May 2015, the patient underwent autologous stem cell transplant. Seven months later, he relapsed again with regions of terminal ileum, appendix, liver, mesentery lymph nodes, left ilium, left femur, as well as axillary and cervical lymph nodes involvement. After a following palliative therapy (thalidomide and rituximab) and 1 cycle of GDP treatment (Fig. 1A), the patient received autologous CART19 expressing murine anti-CD19 scFv and 4–1BB - CD3ζ costimulatory activation domains after fludarabine and cyclophosphamide lymphodepletion chemothrapy (ChiCTR-OCC-15007008). He suffered from a grade 2 CRS and obtained rapid remission (Fig. 1B) but developed spontaneous terminal ileal perforation 38 days following CAR-T infusion. An exploratory laparotomy was performed and the perforation was subsequently repaired.

Five months after CAR-T treatment, the patient complained about fatigue, decreased body weight, bone pain, abdominal pain, and distension. He soon was confirmed a relapse by follow-up PET/CT scan with the involvement of cervical lymph nodes, clavicle, mediastina, left lung hilar, retroperitoneum, intermesenterium, and pelvic cavity (Fig. 1C). The CAR-T cell amount in peripheral blood (PB) was evaluated by flow cytometry,^[2]^ and a percentage of 0.08% meant CAR-T cells decreased dramatically. A mass biopsy in the neck was performed and showed DLBCL cells expressing CD19 antigen, without local CAR-T cell infiltration.

**Figure 2:**
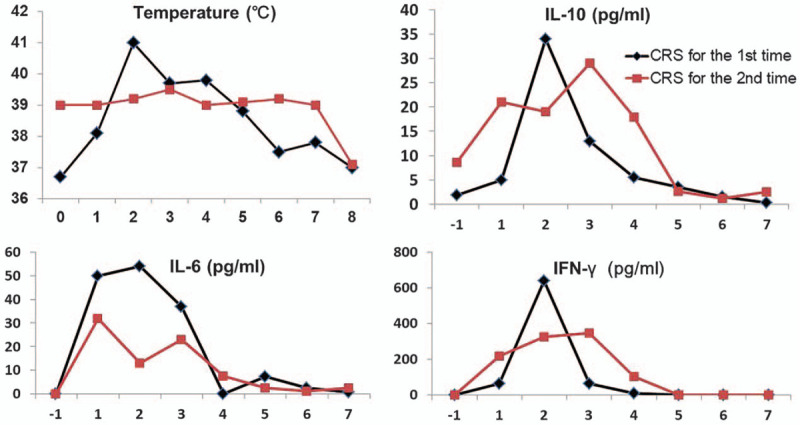
Comparison of the temperature and serum cytokine concentrations between 2 times of CRS. High temperature and increased levels of serum cytokines (IL-10, IL-6, and IFN-γ) can be found at both times of CRS. CRS = cytokine release syndrome.

Then, a second anti-CD19 CAR-T therapy was planned with deliberation. The patient received lymphodepletion chemotherapy with fludarabine (25 mg/m^2^, d1–d3) and cyclophosphamide (500 mg/m^2^, d2–d3). Surprisingly, on the day FC infusion was completed, he quickly developed persistent fever with a highest temperature of 39.5 °C. Checking serum cytokines found a remarkable elevation of IL-6, IL-10, and IFN-γ, even without new CART19 infusion (Fig. 2). The peak serum levels of IL-6, IL-10, and IFN-γ were 32, 29, and 345 pg/mL, respectively. The high fever and serum cytokine levels were similar with his CRS during the first time of CAR-T therapy (Fig. 2). Routine blood cultivations and specific virus DNA detections excluded known bacterial, viral, and fungal infections. And finally a Grade 2 CRS was confirmed.^[8]^ Meanwhile, the analysis by flow cytometry on anti-CD19 CAR-T/CD3+ T cell percentage in PB showed a sudden rise from 0.08% to 19.8%. As for CAR DNA copies by qPCR analysis after FC chemotherapy,^[2]^ there were 438 copies/μg DNA in PB while 2950 copies/μg DNA in mass aspiration of the cervical lymph node. Due the occurring CRS situation, the second CAR-T cell infusion was canceled. The patient suffered from fever for 5 days and slowly recovered with supportive care and without tocilizumab or steroids administration.^[8]^ During fever, his lymphoma-associated symptoms disappeared gradually. By day 9 a serum cytokine analysis showed IFN-γ and IL-6 concentrations decreased to normal ranges. About 20 days after the 2nd CRS, a PET/CT scan showed obvious improvement with largely reduced lymphoma involvement (Fig. 1D).

Unfortunately, 1 month later the patient was complicated with fever and bone pain again. A PET/CT scan showed diffused signs of lymphoma involvement, implying a disease progression (Fig. 1E). Bone biopsy confirmed DLBCL infiltration and an immunophenotypic analysis by flow cytometry showed CD19 dim expression on lymphoma cells (Fig. 1G) as compared with those before FC lymphodepletion chemotherapy (Fig. 1F). The disease was not controlled by salvage chemotherapy and the patient died due to multiple organ failure 1 month later.

## 3 Discussion and conclusion

In current study, we administrated FC chemotherapy for a relapsed DLBCL patient after CAR-T therapy and demonstrated that FC chemotherapy induced pre-existing CAR-T cell revitalization in vivo. The revitalized CAR-T cells eradicated relapsed lymphoma cells and achieved partial remission (PR). For patients relapsed after CAR-T therapy, subsequent therapeutic options might include salvage chemotherapy, a second CAR-T therapy and clinical trials with novel drugs. For this patient, we planned to conduct a second CAR-T therapy since he relapsed with CD19 positive tumors after the first CAR-T therapy. Based on the low percentage of CAR-T cell in his peripheral blood, the cause of disease relapse might be attributed to CAR-T cell exhaustion. After induction with an FC lymphodepletion chemotherapy, the patient experienced CAR-T cell revitalization and a predominant grade 2 CRS. His CAR-T cell percentage increased remarkably in PB and mass biopsy. Then the patient achieved PR but relapsed again with CD19 dim expression. A second CAR-T therapy ceased considering that new CAR-T cells might increase CRS risk and severity, and induce unexpected consequences. Finally the patient suffered from disease progression and a salvage chemotherapy failed to control a CD19-dim dominated relapse.

Recently, Chong et al^[11]^ reported a case in which a programmed death 1 (PD-1) blocking antibody was administered to a patient with refractory DLBCL and progressive lymphoma after CAR-T therapy. Following PD-1 blockade, the patient had a clinically significant antitumor response with an expansion of CAR-T cells. Antibodies blocking the PD-1 receptor on T cells cause tumor regression in multiple cancers by disrupting the PD-L1/PD-1 immune inhibitory axis. That case implied CAR-T cells could be reactivated in vivo under circumstances by disturbing immune equilibrium. In our case, following FC chemotherapy, CAR-T cells were also reactivated and bore antitumor effects. The mechanisms of CAR-T cell reactivation by FC chemotherapy remain unknown. Disturbances on overall immune status by FC might provide insights for elucidation of underlying mechanisms.

Turtle et al^[12]^ demonstrated that in some patients after CAR-T treatment the loss of CAR-T cells was due to the development of CD8+ T cell immunity to the CAR transgene product because of the murine-derived scFv. The immunologic barrier to cells expressing foreign proteins represents a challenge for CAR-T therapy. In our case, CARs incorporate a murine scFv which might in part contribute to the loss of CART19. High-dose post-transplantation Cy (PT/Cy) is an attractive approach for crossing HLA barrier in allogeneic HSCT. One of the important mechanisms is that the replicative DNA synthesis renders alloreactive proliferating T cells uniquely sensitive to Cy.^[13]^ Similarly, alloreactive proliferating T cells to murine scFv are sensitive to Cy, which may partially explain CAR-T cell revitalization in our case.

After FC-induced CRS, the patient showed obvious improvement with reduced lymphoma burden even still with residual lymphoma compromization, however, the residual lymphoma progressed quickly. As demonstrated by mass biopsy, relapsed lymphoma cells had dim expression level of CD19 as compared with lymphoma cells before lymphodepletion chemotherapy, while CD19 is the target of CAR-T cells in our case. Up to date, frequencies and subsequences of CD19 positive and CD19 dim or negative relapse are still unclear.^[14,15]^

Together, our clinical observation and correlative laboratory findings demonstrated that FC chemotherapy revitalized CAR-T cells in vivo in a patient with relapsed DLBCL. Revitalized CAR-T cells facilitated to achieve PR with CRS, and the patient died of PD presumably due to a CD19-low dominant relapse. CAR-T therapy is still confronted with huge challenges in clinical practice. Our study supports the application of opitimized bi-specific CARs in initial CAR-T treatment and indicates an urgent demand of novel strategies for relapsed malignancies after CAR-T therapy.

## Author contributions

**Analysis and interpretation of data:** Lei Xiao, Jiazhen Cui, Zhao Wu, He Huang.

**Patient data collection/Data acquisition:** Yongxian Hu, Zuyu Liang, Hao Zhang, Mi Shao, Guoqing Wei, Qu Cui, Wenjun Wu.

**Writing and/or critical revision of the manuscript:** Yongxian Hu, Qu Cui, He Huang.
